# Mdm2 targeting via PROteolysis TArgeting Chimeras (PROTAC) is efficient in p53 wildtype, p53-mutated, and abemaciclib-resistant estrogen receptor-positive cell lines and superior to mdm2 inhibition

**DOI:** 10.1186/s12885-025-14361-z

**Published:** 2025-06-01

**Authors:** Alina Goerg, Gerhard Piendl, Veruschka Albert, Olaf Ortmann, Anja Kathrin Wege, Gero Brockhoff

**Affiliations:** 1 Department of Gynecology and Obstetrics, University Medical Center Regensburg, Franz-Josef-Strauß-Allee 11, 93053 Regensburg, Germany; 2 Bavarian Cancer Research Center (BZKF), Regensburg, Germany

**Keywords:** Luminal breast cancer, Estrogen receptor-positive breast cancer, mdm2, p53 mutation, Abemaciclib, PROTAC

## Abstract

**Purpose:**

The human double minute 2 homolog hdm2, alias mdm2, is the main negative-regulator of the tumor suppressor p53. In that capacity, mdm2 is a promising but not yet utilized molecular target for the treatment of breast cancer, however, its inhibition by small molecules is rather inappropriate. Instead, mdm2 degradation by PROteolysis TArgeting Chimeras (PROTAC) is expected to be highly specific, to exhibit pronounced efficiency and minimal side effects. Moreover, there is profound evidence that mdm2-specific PROTAC degraders are efficient even in tumor cells harboring p53 loss-of-function mutations.

**Methods:**

We comparatively treated p53 wildtype / abemaciclib-sensitive and -resistant MCF-7, as well as p53-mutated T-47D estrogen receptor-positive breast cancer cells in-vitro with the mdm2 inhibitor AMG-232 and an mdm2 PROTAC degrader. The molecular signaling as a function of mdm2 inhibition and degradation was assessed and cell viability and cell cycle kinetics were monitored. In addition, potential PROTAC effects on the expression of immune-related markers MHC-I, MHC-II, PD-L1, PD-L2, and CD276 were determined.

**Results:**

PROTAC treatment considerably attenuated cell proliferations and was superior to mdm2 inhibition in p53 wildtype and even in p53-mutated cells. Proliferation-associated pathways were significantly but differentially affected, including p73, retinoblastoma protein, and the transcription factor E2F1. MHC-I and CD276 were significantly downregulated.

**Conclusion:**

The data reveal deeper insight into PROTAC-induced molecular mechanisms in luminal breast cancer cells with and without p53 mutations. The study provides the basis to evaluate the therapeutic applicability of anti-mdm2 PROTAC degraders in an appropriate preclinical in-vivo setting, for example in humanized tumor mice.

**Supplementary Information:**

The online version contains supplementary material available at 10.1186/s12885-025-14361-z.

## Introduction

The E3 ubiquitin-protein ligase named mouse double minute 2 homolog (mdm2, less frequently specified as human double minute 2 homolog, hdm2) is an essential negative regulator of protein 53 (p53), commonly referred to as the “main guardian of the genome” [1]. The tumor suppressor p53, specified by a molecular weight of 53 kDa, acts primarily as a transcription factor and thereby controls cell proliferation, initiates the repair of DNA damages, and maintains the balance between cellular survival and programmed cell death [2].

The main function of the E3 ubiquitin ligase mdm2 is the labeling of proteins dedicated to undergo proteasomal degradation. Protein ubiquitination is an oligo-step mechanism that involves the ATP-driven transfer of ubiquitin across a cascade of specific ligases named E1, E2, and finally E3. One of the best investigated E3 ligases is mdm2 that enables the ubiquitination of p53 (and other molecules) and thereby seals p53 fate towards proteasomal elimination [3]. In turn, reduced presence or even the absence of (wildtype) p53 enables uncontrolled cell proliferation and thus malignant cell growth. Mutations within the p53 gene locus (i.e., TP53) are most often single nucleotide changes that cause p53 loss of function [4]. With an overall frequency of 60%, p53 mutations are highly relevant in breast cancer (BC) [5] and the prevalence of p53 mutations is about 20% in primary luminal (i.e., estrogen receptor-positive, ESR) BC, 70% in triple negative, and 40% in HER2-enriched BC [6].

Mdm2 not only controls p53 but also auto-regulates its own expression. A restrained auto-ubiquitination enables controlled auto-degradation. Another mechanism involved in maintaining the mdm2 expression is an autoregulatory feedback loop executed by p53 since one of the diverse transcription targets of p53 is mdm2 [7]. As an oncogene/oncoprotein, mdm2 can initiate carcinogenesis and potentially drives uncontrolled tumor growth. Enhanced mdm2 expression and/or an enriched gene copy number has been found in a variety of malignancies with different frequencies [8, 9]. Pronounced mdm2 gene copy numbers in BC have been reported probably at first in 1995 [10] and were found in a cohort of > 2,000 BCs already two decades ago [11]. Our group identified mdm2 gene amplification in luminal BC with an overall frequency in luminal A and B BCs of 10.4% [12]. In this and other studies mdm2 gene amplifications and p53 loss-of-function mutations have been found to mostly occur exclusively [8], however, both alterations affect the same signaling pathway [3] and are considered to have equivalent consequences. Nevertheless, pronounced mdm2 expression observed in BC is not necessarily associated with an mdm2 gene amplification [13].

The frequency of ESR and/or progesterone-receptor-positive BC is about 70% of all BCs [5, 6]. The anti-CDK4/6 treatment against Cyclin Dependent Kinases 4/6 (CDK4/6) (commonly in combination with an endocrine therapy) is nowadays a mainstay for the treatment of this BC subtype. Previous therapy approvals for the use of CDK4/6 inhibitors (CDK4/6i), namely abemaciclib (Verzenios™), palbociclib (Ibrance™), ribociclib (Kisquali™) by the U.S. Food and Drug Administration (FDA) and the European Medicines Agency (EMA) were primarily based on various MONARCH, PALOMA, and CORALEEN studies [14]. However, an insufficient therapy response in the presence of CDK4/6i or tumor progression and relapse is not uncommon.

Upon interaction with Cyclin-D1 and in its active (i.e., phosphorylated) state CDK4/6 facilitates the cell cycle progress by phosphorylation of the retinoblastoma (Rb) suppressor protein, which is followed by the release and activation of the transcription factor E2F(1). This mechanisms enables the G1-S-phase transition.

A large number of molecules involved in these processes have been associated with anti-CDK4/6 resistance [15–18], however, none of them became clinically (therapeutically) relevant yet.

Beyond the canonical CDK4/6-dependent signaling mdm2 is a “hot” but widely underestimated candidate for indicating an anti-CDK4/6 therapy success. A decelerated cell cycle progress or cellular quiescence seen in anti-CDK4-treated cells, however, is basically reversible and is likely to reverse towards proliferation. In contrast, the inactivation / downregulating of mdm2 under anti-CDK4/6 treatment signifies cellular senescence, which is essential to irreversibly block cell proliferation and precedes target cell elimination [19–21]. Thus, mdm2 does not only have the capacity to indicate either success or failure to an anti-CDK4/6 therapy but might also represent a substitute or additional target to treat ESR-positive BC. Targeting mdm2 could be done alternatively or in combination with CDK4/6i [22, 23]. This consideration is substantiated by our recent findings that an increased gene copy number of mdm2 in luminal BC serves as a negative prognosticator for the course and outcome of disease [12, 24].

Independently of CDK4/6, numerous mdm2 inhibitors have been developed in the past, however, none of them achieved clinical applicability [25–29]. This is mainly due to three reasons: First: Inhibitors unspecifically inhibit off-target molecules (amongst them other ubiquitin ligases). Second: Inhibitors cause severe side effects when used in (pre)clinical trials. Third: Inhibitors are entirely ineffective in tumor cells with p53 loss-of-function mutations (see above). Thus, other strategies are required to therapeutically address mdm2, with and without consideration of an anti-CDK4/6 targeting. Against this background, the use of PROteolysis TArgeting Chimeras (PROTAC) represents a promising strategy. PROTAC are heterobifunctional molecules consisting of two ligands joined by a linker [30, 31]. One ligand binds an E3 ubiquitin ligase whereas the other recruits a protein of interest (POI). The ternary complex consisting of the ubiquitin ligase, the PROTAC molecule, and the POI enables the highly-specific degradation of the target molecule (i.e., the POI). Remarkably, to the mechanism of action, PROTACs proved to be useful to tackle previously undruggable targets [31].

Here we used a highly specific, double Nutlin-3-based PROTAC to target mdm2 in native, p53 wildtype MCF-7 (here MCF-7nat), abemaciclib-resistant MCF-7 (MCF-7res), and p53-mutated T-47D BC cells. The E3-ubiquitin ligase represents the effector of a PROTAC molecule and is dedicated to degrade the protein of interest (POI). Basically, a variety of E3-ubiquitin ligases can be used to design/synthetize a PROTAC molecule. Presumably, Cereblon (CRBN)-based or Von Hippel-Lindau tumor suppressor (vhl)-based PROTACs are being prevalently used. However, CRBN-based PROTACs, are known to potentially exhibit unwanted effects as a “molecular glue” [32]. For this study, a double-Nutlin based PROTAC that recruits mdm2 for both the target and the effector molecule was applied. This PROTAC has been shown to be efficient in non-small lung cancer [33] and other malignant cells [34–36]. However, the application to ESR-pos. BC cells has not been evaluated, yet.

MCF-7nat, MCF-7res, and T-47D BC cells reflect the ESR-positive BC type with different, treatment-relevant characteristics. The proprietarily synthesized anti-mdm2 PROTAC (in the following just named PROTAC) has been designed to enable mdm2 self-degradation [33]. Thus, mdm2 represents both the degrader and the POI. We hypothesized a superior PROTAC treatment efficiency over the use of AMG-232 (Navtemadlin, a selective p53-mdm2 inhibitor, not a degrader).

Proliferation kinetics, as a decisive parameter of treatment efficiency, were quantitatively assessed by flow cytometry as a function of PROTAC and AMG-232 treatment. In addition, the induction of apoptotic cell death, regulation of immune-associated molecules, and the proliferation- and survival-associated intracellular signaling upon treatment were investigated.

This study is the first-time report on the treatment efficiency and underlying molecular mechanism initiated by the use of PROTAC in luminal, p53 wildtype, and mutated cells.

## Materials and methods

### BC cell lines, cell culture, and cell treatments

All cell lines used in this study were authenticated by the German Collection of Microorganisms and Cell Cultures GmbH (DSMZ, Braunschweig, Germany). ESR positive human BC cell lines MCF-7 (American Type Culture Collection no. HTB-22™, RRID: CVCL_0031), T-47D (ATCC no. HTB-133™, RRID: CVCL_0553) were cultivated in DMEM supplemented with 5% fetal bovine serum (Thermo Fisher Scientific) at 37 °C and 5% CO_2_. T-47D cells harbor a p53 loss of function mutation (mtp53 L194F), whereas MCF-7 cells are p53 wildtype (wt). Abemaciclib resistant MCF-7 (MCF-7res) cells were generated by continuous cell exposition to gradually increasing (1–100 nM) abemaciclib concentrations [24]. “PROTAC mdm2-degrader-2” was purchased from MedChemExpress (NJ 08852, USA). PROTAC treatments were done with concentrations raising from 2.5 to 20 µM according to the respective experiment. AMG-232 was applied at a previously evaluated concentration of only 100 nM [12, 24]. Both PROTAC and AMG-232 were solved in DMSO.

### Dynamic proliferation assessment by flow cytometry

Flow cytometric BrdU/Hoechst quenching measurements (well established and is being frequently used in our lab for many years) were performed as described previously in more detail [24, 37–40]. In brief, cells seeded at appropriate cell densities were continuously exposed to 20 µM bromodeoxyuridine (BrdU) and 2‘deoxycytidine (DC) (both reagents from Sigma-Aldrich, Merck, Darmstadt, Germany) at half-equimolar concentration. Instead of thymidine, BrdU gets incorporated into the DNA while an imbalanced nucleic acid metabolism is prevented in the presence of DC. As evaluated in advance, the supplementation with only 20 µM BrdU did not affect the cell proliferation (doubling times) of the three cell lines used. Cells were harvested in intervals covering a time range up to 96 h. Potentially apoptotic cells in the supernatant were discarded. After detachment, the cells were stored at -20 °C at a concentration of 10^6^ cells/ml in freezing medium (DMEM medium + 20% FCS 7.5% dimethyl sulfoxide [DMSO]) until flow cytometric analysis. For cell staining, thawed cells were washed twice with 2 ml of ice cold DNA-staining buffer (100 mM Tris-HCl, pH 7.4, 154 mM NaCl, 1 mM CaCl_2_, 0.5 mM MgCl2, 0.1% IGEPAL CA-630 [Nonylphenylpolyethyleneglycol], 0.2% BSA). 5 × 10^5^ cells were resuspended in 1 ml buffer supplemented with 40 g/ml (2–4 Units/ml) RNase and 1.2 µg/ml Hoechst 33258 (Sigma-Aldrich) and incubated for 15 min at 37 °C. Cellular DNA was additionally stained with propidium iodide (1.5 µg/ml) for 15 min on ice. Flow cytometric measurements were done using a FACSCanto-II (BD Biosciences, San Jose, CA) equipped with three lasers (standard optical configuration). Samples were measured using FACSDiva Software v7.0 software (BD Biosciences). 50,000 events / sample were collected. Data were analyzed with FlowJo software v10.8 (BD Biosciences, San Jose, CA, USA). Supplement Fig. 1 illustrates the course of a non-synchronized cell population and subcohorts within three successive cell cycles (red 1st, color 2nd, green 3rd) monitored within a period of 96 h. Based on this pattern, cell subcohorts can be attributed to individual cell cycle and individual cell cycle phases. An appropriate gating allows to quantify percentages of individual cell fractions.

### Flow cytometric evaluation of apoptotic cells death via Annexin-V staining and Propidium iodide exclusion

For the analysis of apoptosis, adherent cells and detached cells in the supernatant were harvested, pooled and suspended in 75 µl Annexin-V-FITC solution (Immunotools, Friesoythe, Germany) containing 5 µl Annexin-V-FITC and 70 µl binding buffer (0.1 M HEPES). After 20 min of incubation on ice in the dark, the cells were centrifuged and resuspended in 200 µl binding buffer. Prior to the measurement, the DAPI dye (Sigma-Aldrich, Merck, Darmstadt, Germany) was added to a final concentration of 0.1 µg/ml. Measurements started less than 3 min after the addition of DAPI.

### Immune marker phenotyping by flow cytometry

Cells for flow-cytometric immune marker phenotyping were harvested from cell culture using standard procedures as described by our group previously [41]. Antibodies against the following antigens/markers and conjugated with following fluorochromes were used and incubated for 30 min at 4 °C in the presence of 250,000 cells, respectively: MHC I-PE (Thermo Fisher Scientific Cat# MA1-10346, MEM-123, RRID: AB_11154825), PD-L1-BV421 (BioLegend Cat# 329714, 29E2A3, RRID: AB_2563852), MHC II-FITC (Biolegend Cat# 361706, Tü39, RRID: AB_2563192), PD-L2-PE/Cyanine 7 (Biolegend Cat# 345512, MIH18, RRID: AB_2687280), CD276-APC (Biolegend Cat# 351006, MIH42, RRID: AB_2564404) Furthermore, for the evaluation of live and dead cells, cells were stained with a Zombie NIR™ Fixable Viability Kit (Biolegend Cat# 423105) At least 30,000 cells were collected and evaluated with respect to the percentage of positive cells and their marker expression levels.

### Protein isolation and western blotting

Total protein isolation was performed on ice using cell lysis buffer (Cell Signaling Technology) supplemented with Halt™ Protease (Thermo Fisher Scientific) and Phosphatase Inhibitor Cocktail (Carl Roth). The protein concentration was quantified using the Pierce BCA Protein Assay Kit (Thermo Fisher Scientific). According to protein size, 20 µg protein per lane was separated in 10 or 15% SDS-PAGE under reducing conditions (mercaptoethanol) and subsequently blotted onto polyvinylidene difluoride (PVDF) membranes. The membranes were then blocked in TBS-T buffer 5% BSA or 5% low-fat milk and 1% Tween for 1 h. Only for the usage of the MDM2 antibody, the blocking time was extended to 3 h. After that, the primary antibodies were incubated in 5% BSA or 5% low-fat milk overnight with a concentration of 1:1000 if not otherwise specified.

The following primary antibodies were used and obtained from Cell Signaling Technology: MDM2 (Cat# 86934, RRID: AB_2784534), p53 (Cat# 9282, RRID: AB_331476), Rb (Cat# 9309, RRID: AB_823629; 1:2000), phospho-Rb Ser608 (Cat# 8147, RRID: AB_10949974), phospho-Rb Ser780 (Cat# 8180, RRID: AB_10950972), phosho-Rb Ser795 (Cat# 9301, RRID: AB_330013), phospho-Rb Ser807/811 (Cat# 8516, RRID: AB_11178658), E2F1 (Cat# 3742, RRID: AB_2096936), p21 (Cat# 2947, RRID: AB_823586). The p73 antibody was purchased from Abcam (Cat# 40658, RRID: AB_776999). Anti-actin antibody was used as a loading control (Sigma-Aldrich Cat# A2066, RRID: AB_476693; 1:5,000). After incubation overnight and washing of the membranes, secondary antibodies anti-mouse (Cat# 7076, RRID: AB_330924) and anti-rabbit (Cat# 7074, RRID: AB_2099233), both Cell Signaling Technology, were incubated for 1 h at room temperature, diluted 1:2000. PageRuler plus prestained protein ladder (Thermo Fisher Scientific) was utilized as a protein size standard. For protein visualization, chemiluminescent substrate SuperSignal™ west pico PLUS (Thermo Fisher Scientific) and ChemiDoc Imaging System (Image Lab 6.0.1, BioRad, RRID: SCR_014210) were used.

### Statistical analyses

The statistical analyses were done using GraphPad Prism (RRID: SCR_002798), version 6. Two-way ANOVA, or One-way ANOVA and Sidak’s or Dunnett’s or Tukey’s multiple comparisons test were performed as indicated in the figure legends. Differences with a *p*-value ≤ 0,05 were considered significant. Moreover, the significance was further classified depending on the strength of significance: not significant (ns) = *p* > 0,05; * *p* = ≤ 0,05; ** *p* = ≤ 0,01; *** *p* = ≤ 0,001; **** *p* ≤ 0,0001. Data are presented as mean with standard deviation (SD) unless otherwise described.

## Results

### The inhibition of MCF-7 and T-47D cells by PROTAC is efficient and superior to AMG-232 treatment

Proliferation kinetics were assessed by flow cytometry to evaluate and to compare the treatment efficiencies of the mdm2 inhibitor AMG-232 and PROTAC on of MCF-7nat, MCF-7res, and T-47D cells. The approach enables to exactly determine the treatment effect on the cell cycle progress and reveals first clues for the mechanisms of action caused by the individual drugs.

**Fig. 1:**
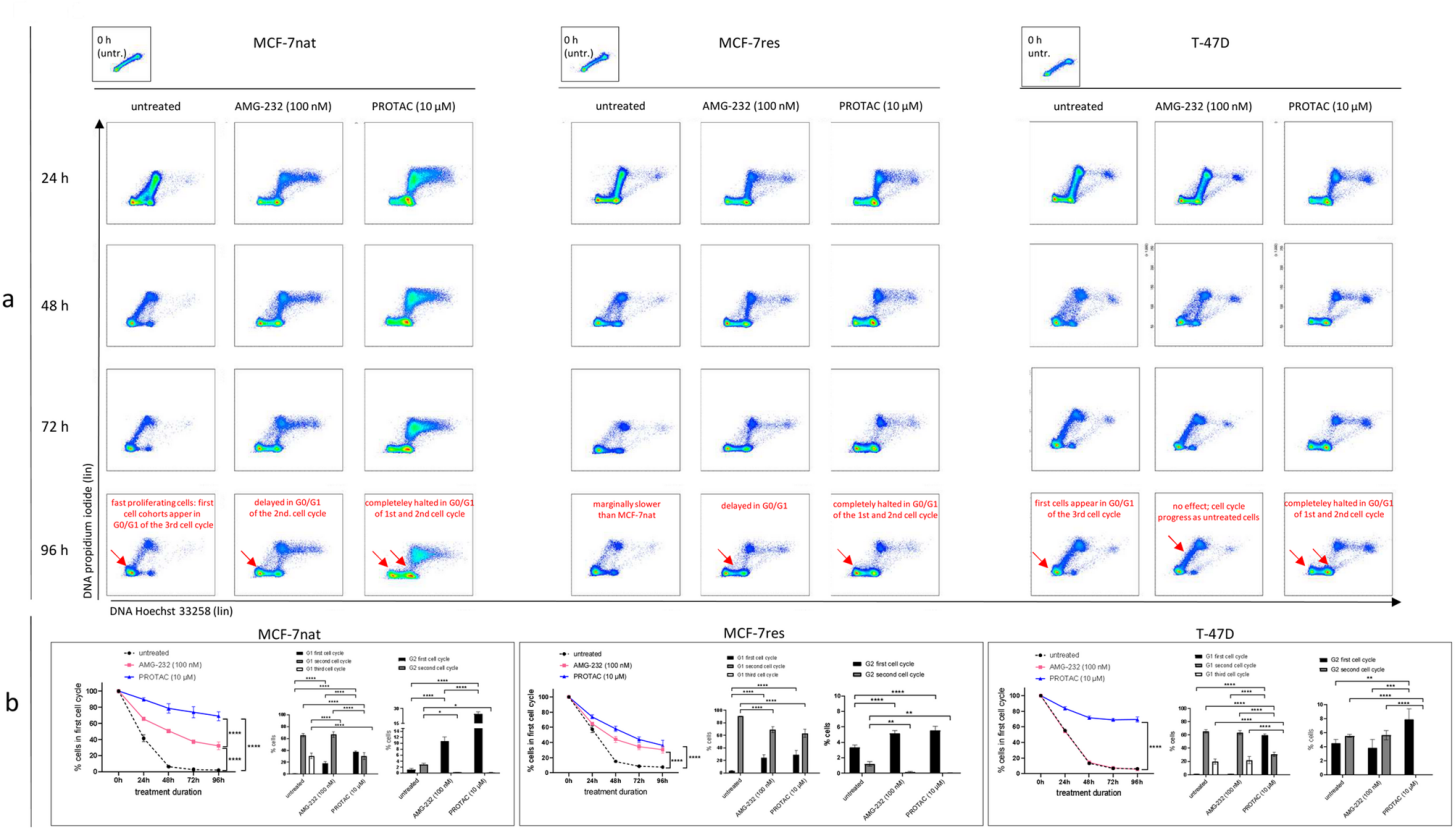
**a**: Cell cycle progression of MCF-7nat, MCF-7res, and T-47D cells within a period of 96 h as a function of AMG-232 and PROTAC treatment and compared to untreated cells. Flow cytometric measurements were done in 24, 48, 72, and 96 h time intervals. Annotations and arrows in red indicate the most pronounced treatment-induced effects. **b**: G1-phase (first cell cycle) exit curves of MCF-7nat, MCF-7res, and T-47D cells. Percentages of cells attributed to the first cells cycle over a period of 96 h (x-axis) as a function of treatments are displayed. Cell cohorts within the first cell cycle were calculated as a subfraction of the total cohort. This calculation differentiates between cell cohorts of G0/G1-phases of the first, second, and third cell cycle. Bar graphs shown in the very right postion of subpanel b, respectively, show cell cohorts of G2/M-phases of the first and second cell cycle. Statistics was done by applying Two-way ANOVA and Tukey’s posthoc test for multiple comparisons. * *p* ≤ 0,05; ** *p* ≤ 0,01; *** *p* ≤ 0,001; **** *p* ≤ 0,0001

Figure 1a gives an overview of the cell cycle progress of MCF-7nat, MCF-7res, and T-47D cells within a period of 96 h as a function of AMG-232 and PROTAC treatment. Main effects / inhibitions are highlighted by red arrows and annotated accordingly. Cell subcohorts were calculated based on the scheme given in supplement Fig. 1. After 96 h, the first untreated MCF-7nat cells enter the G1-phase of the third cell cycle. In contrast, total cell cohorts are totally halted in G0/G1 when exposed toed to PROTAC, whereas the majority of AMG-22-teated cells are just in G0/G1-phase of the second cell cycle. MCF-7res show a similar response as the native cells, however, they proliferate slower. T-47D cells are completely resistant to AMG-232 treatment, however, show pronounced sensitivity when exposed to PROTAC.

The colored graphs in Fig. 1b show exit curves derived from the first cell cycle quantified by appropriate gating [24]. Generally, higher values indicate a stronger inhibition of cell progression to the next phase of the cell cycle or the next cell cycle phase. Compared to untreated cells, AMG-232-treated MCF-7nat cells experience a significant delay of cells exiting from the first cell cycle (40% do not progress within 96 h) and partially arrest in G2-/M-phase of the first cell cycle in general. The exit from the first cell cycle is even more substantially delayed in the presence of PROTAC: 80% of the cells were not able to enter the second cell cycle within 96 h and in contrast to AMG-232-treated cells, not even the smallest cells fraction could enter the third cell cycle in the presence of PROTAC within the observation time. Unexpectedly, MCF-7res cells show similar sensitivity to AMG-232 and PROTAC treatment: In both cases a cell cohort of 40% did not left the first cell cycle. T-47D cells were insensitive to AMG-232 treatment but highly responsive to PROTAC treatment. The middle graph in each box of Fig. 1b displays an advanced dissection for the cell cycle by the differentiating of cell subcohorts in G0/G1 of the first, second and third cell cycle. PROTAC-treated MCF-7nat cells show the largest cell cohorts in G0/G1 of the first cell cycle (38%) vs. AMG-232-treated cells (20%). By comparison, most MCF-7res cells are halted in G0/G1-phase of the second cells but not the first cell cycle. The cell cohort distribution of AMG-232-treated T-47D cells does not differ from untreated cells, while 60% of PROTAC-treated cells are efficiently halted in G0/G1 of the first cell cycle. The very right graph in each box quantitatively displays cells, which were additionally halted in G2/M-phase of the first or second cell cycle. A pronounced additional block (30%, potentially an arrest) can be seen in PROTAC-treated MCF-7nat cells. This additional effect can be observed in T-47D, but not in MCF-7res cells.

### PROTAC treatment causes apoptotic cell death in MCF-7nat, MCF-7res and T-47D cells with a similar time course

A flow cytometric Annexin-V/DAPI assay was applied to quantify the extent of PROTAC-induced cell death at two different points in time (24 and 96 h) and using two different PROTA concentrations (5 and 10 µM). Like proliferation measurements, the approach delivers kinetic information and allows to determine a potential cytotoxic effect on of MCF-7nat, MCF-7res, and T-47D cells.

**Fig. 2:**
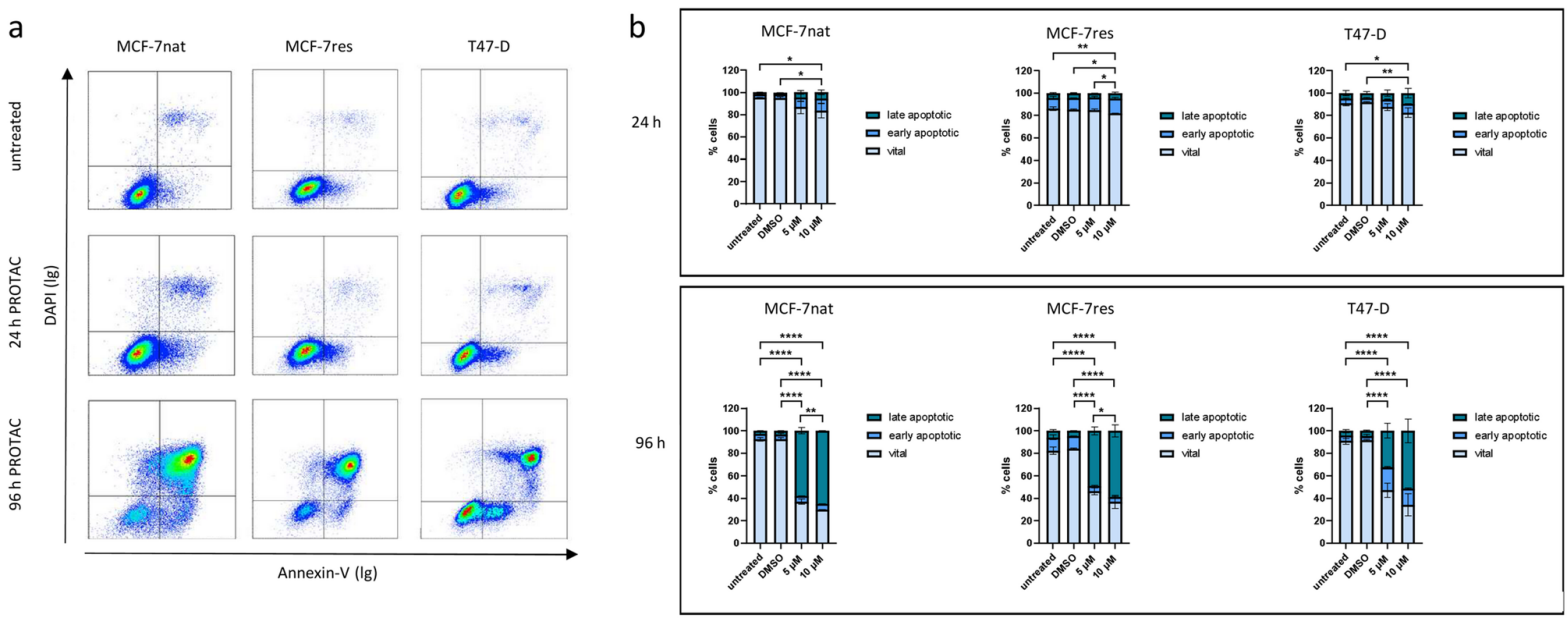
**a**: Examples of flow cytometric DAPI/Annexin-V measurements of MCF-7nat, MCF-7res, and T-47D cells 96 h after PROTAC treatments are shown. Annexin-V-neg./DAPI-neg. cells are considered vital, Annexin-V-pos./DAPI-neg. cells are early apoptotic, and Annexin-V-pos./DAPI-pos. cells are late apoptotic. **b**: Absolute fractions of vital, early apoptotic, and late apoptotic MCF-7nat, MCF-7res, and T-47D cells as a function of treatment with 5 and 10 µM PROTAC are given in bar charts. Flow cytometric measurement were done after 24 (upper row) and 96 h (lower row9 of treatment. Statistics was done by applying Two-way ANOVA and Tukey’s posthoc test for multiple comparisons. * *p* ≤ 0,05; ** *p* ≤ 0,01; *** *p* ≤ 0,001; **** *p* ≤ 0,0001

Figure 2 shows the effect of PROTAC treatments (5 or 10 µM) on cell viability. Panel a exemplifies flow-cytometric measurements done after 24 and 96 h after. Panel b: Quantitative data of the apoptotic process are given. The fraction of apoptotic cells after 24 h was quite small in all three cells types and did not exceed 20% when 10 µM PROTAC was applied. However, after 96 h the total apoptotic cell fractions were found in the range of 60% (5 µM PROTAC) to 70% (10 µM PROTAC) for all three cell types (MCF-7nat, MCF-7res, T-47D). The generation of apoptotic cells by PROTAC treatments was similar in all cell types, including the p53-mutated cells.

### Decrease of mdm2 and p53 in T-47D cells but mdm2 stabilization in MCF-7 upon PROTAC treatment with cell line specific downstream signaling alterations

MCF-7nat, MCF-7res, and T-47D cells were exposed to 2.5–20 µM PROTAC in order to determine the lowest concentration that shows the maximum effect on the primary (i.e., mdm2) and secondary molecular target (i.e., p53). Concentrations were applied in conformity with the studies published by He et al. and Adams et al. [33, 42].

**Fig. 3:**
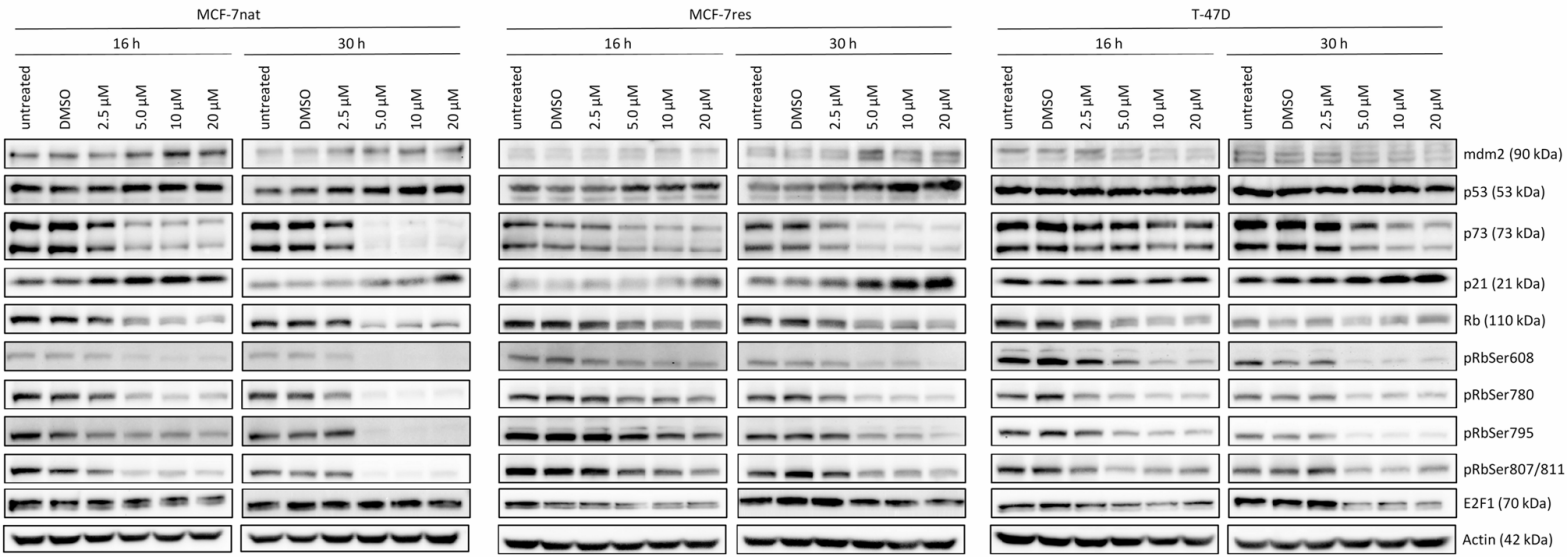
Intracellular signaling in MCF-7nat, MCF-7res, and T-47D cells as a function of PROTAC treatment with increasing concentrations. Cells were treated for 16 and 30 h with 2.5, 5.0, 10, and 20 µM PROTAC before cells lysis and protein isolation. Completely untreated and DMSO-treated (PROTAC solvent) cells served as negative controls. Representative Western Blots of mdm2, p53, p73, p21, E2F1, Rb, pRbSer608, pRbSer780, pRbSer795, and pRbSer807/811 are shown

Figure 3 gives an overview of the mdm2 and p53 expression and downstream signaling molecules as a function of PROTAC treatments applied with increasing concentrations after 16 h and 30 h, respectively. In both MCF-7 cell types the mdm2 expression increased in the presence of PRTOAC concentration-dependently. Simultaneously, the p53 expression raised. In contrast, mdm2 decreased in T-47D cells, while the p53 expression was not affected in this p53-mutated cell line in the presence of increasing PROTAC concentrations. p73 was nearly completely eliminated in MCF-7nat- and MCF-7res cells and considerably reduced in T-47D cells in the presence of 5.0 µM PROTAC upwards. Following downstream signaling, p21 expression considerably increased as a function of increasing PROTAC concentrations in all three cell types. The presence of E2F1 was reduced in MCF-7res and T-47D but not in MCF-7nat cells in the presence of PROTAC. All three cell types have a substantial and gradually more pronounced decrease of Rb as a function of PROTAC concentration in common. Simultaneously, the Rb phosphorylation at four different Rb residues severely declined. For the assessment of proliferation kinetics and for extended molecular analysis we applied 10 µM PROTAC. This concentration was considered to cause maximal specific and minimal off-target effects.

### Regulation of mdm2, p53 und downstream signaling molecules in MCF-7nat, MCF-7res, and T-47D cells by PROTAC treatment of as a function of time

After evaluating the lowest possible but most efficient PROTAC concentration, time-dependent molecular regulation was evaluated within a period of 48 h after single administration.

**Fig. 4:**
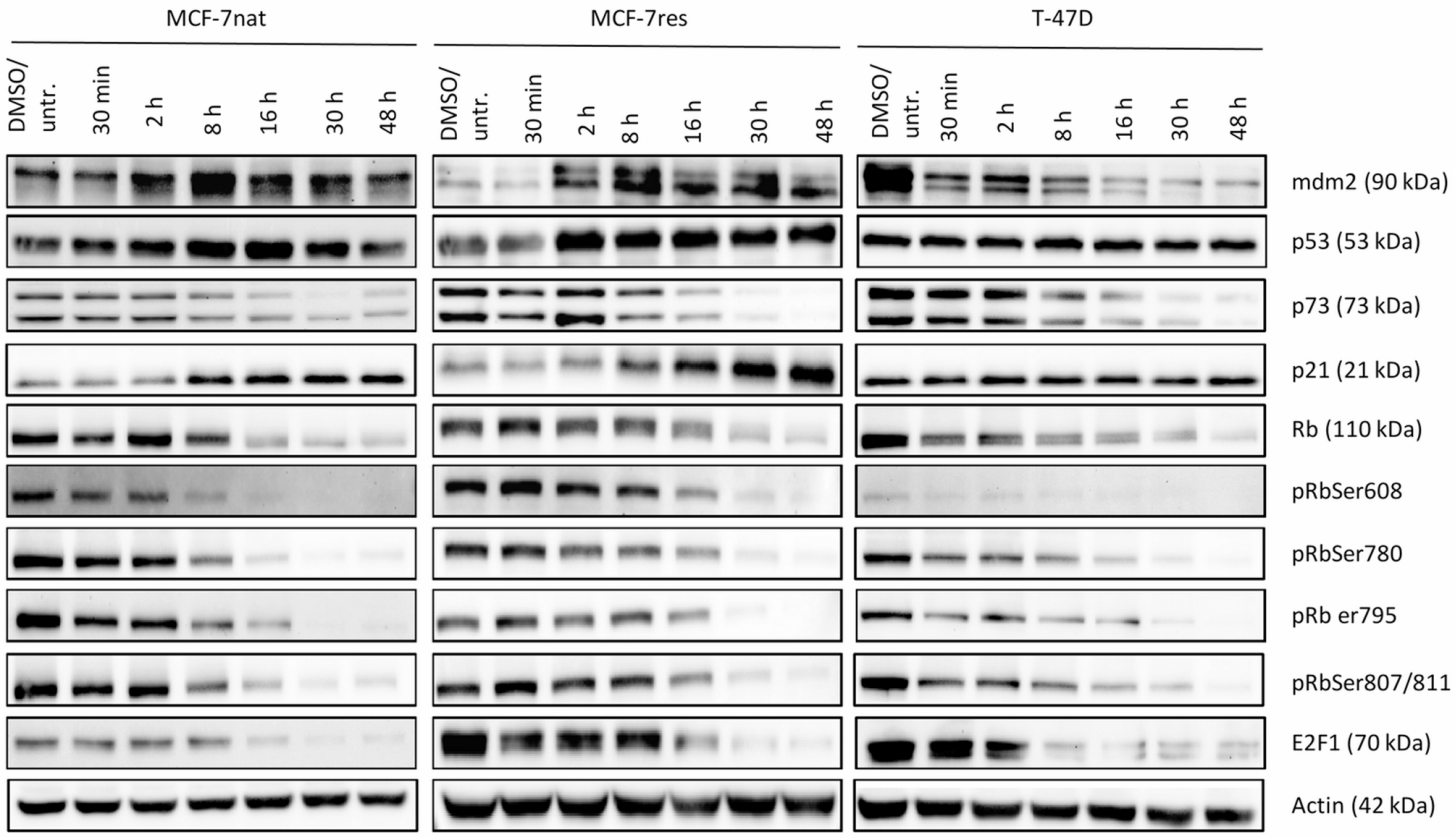
Intracellular signaling in MCF-7nat, MCF-7res, and T-47D cells as a function of AMG-232 treatment over a period of 48 h. Samples taken after 30 min, 2, 8, 16, 30, and 48 h intervals are shown. mdm2, p53, p73, p21, E2F1, Rb, pRbSer608, Ser780, Ser795, and Ser807/811 were analyzed

Western blots in Fig. 4 show the amount of mdm2, p53, and signaling molecules (presence and phosphorylation) in all cell types over time (up to 48 h). The increase of mdm2 in MCF-7nat and MCF-7res cells and the considerable decrease in T-47D cells within the observation time becomes blatantly obvious. p53 transiently increases over time in MCF-7 cells but remained unaffected in T-47D cells. p21 is considerably increased and stabilized in MCF-7 cells over time, however remains unaffected in T-47D cells. p73, E2F1, and Rb, however, decreases time-dependently in all cell types in the presence of PROTAC. Simultaneously, total Rb decreases and Rb phosphorylation at four different protein sites disappear.

### Regulation of mdm2, p53 und downstream signaling molecules in MCF-7nat, MCF-7res, and T-47D cells by AMG-232 treatment as a function of time

Analogous to the PROTAC treatment we determined time-dependent molecular alterations of AMG-232-induced effects on target cells within a period of 48 h after single administration. These analyses allows the direct comparison of molecular mechanisms induced by inhibitors in comparison to degraders.

Western blots in supplement Fig. 2 display the amount of mdm2, p53, and signaling molecules (presence and phosphorylation) in all cell types over time (up to 48 h) as a function of AMG-232 treatment. Likewise PROTAC treatments, mdm2 was upregulated within 2 and 30 h of AMG-232 treatment in both MCF-7 cell types, however this effect was transient. Although mdm2 was considerably downregulated in T-47D cells by the PROTAC treatment (Fig. 4), the exposition of this cell line to AMG-232 caused a slight and very transient (2–8 h) mdm2 increase, which was almost completely reduced to the expression level of untreated cells after 16 h. The most striking difference between PROTAC and AMG-232-treated cells was the substantial decrease of p73 in the presence of PROTAC, whereas p73 was basically unaffected upon AMG-232 treatment (MCF-7nat), just slightly downregulated in MCF-7res, but even upregulated in T-47D cells. Likewise PROTAC treatments, AMG-232-treated cells show an appreciable increase of p21, which is persistent in both MCF-7 cell types but transient in T-47D cells. The expression of Rb is downregulated and the Rb phosphorylations decrease in the presence of AMG-232. The amount of E2F1 substantially declined in MCF-7nat and MCF-7res cells but decreased only slightly and transiently in T-47D cells in the presence of AMG-232.

### Reduced MHC-I and CD276 in MCF-7nat, MCF-7res, and T-47D cells upon PROTAC treatment

In order to determine the effect of PRTOTAC treatment on the immunogenicity of target cells, we quantitatively assessed the expression of MHC-I and MHC-II and the immune checkpoint molecules PD-L1, PD-L2, and CD276 by flow cytometry.

**Fig. 5:**
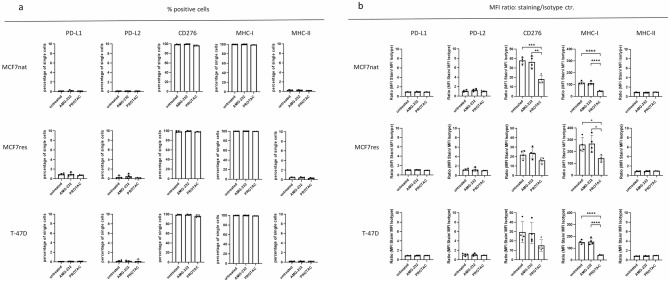
Flow cytometric immune marker phenotyping of MCF-7nat, MCF-7res, and T-47D cells after 24 h AMG-232 (100 nM) and 24 h PROTAC (10 µM) treatment and compared to untreated cells. **a**: The percentages of marker-positive cells (**a**) were quantified based on isotype controls and the relative expression of marker-positive cells. **b**: the extent of marker expression is expressed as mean fluorescence intensity (MFI) as derived from markers-stained cells divided by the MFI of the isotype control. Statistics was done by applying Two-way ANOVA and Tukey’s posthoc test for multiple comparisons. * *p* ≤ 0,05; ** *p* ≤ 0,01; *** *p* ≤ 0,001; **** *p* ≤ 0,0001

Just very low PD-L1, PD-L2, and MHC-II expression levels could be detected in all three cell types, which were not altered in the presence of AMG-232 or PROTAC (Fig. 5, panel a). Instead, about 100% of all three cell types were CD276 and MHC-I-positive. Bar graphs in panel b of Fig. 5 show unit-less MFI values of marker-stainings representing the respective expression densities. The MHC-I expression was significantly reduced in all three cell types upon PROTAC treatment. Likewise, CD276 was decreased in all three cell types as well, which was a significant effect in MCF-7nat cells. In contrast, MHC-I and CD276 (as well as PD-L1, PD-L2, and MHC-II) were not altered when cells were exposed to AMG-232.

## Discussion

Here, we quantitatively assessed proliferation kinetics and the survival capacity of ESR-positive, abemaciclib-sensitive and -resistant MCF-7, as well as p53-mutated T-47D BC cells as a function of PROTAC treatment. For comparison purposes, we exposed the cells to the mdm2 inhibitor AMG-232. The treatment effect on the canonical CDK4/6-Rb-E2F1 and the non-canonical bypass CDK4/6-mdm2-p53-p21 signaling axis were analyzed and associated with respective treatment efficiencies.

Dynamic proliferation analyses and corresponding cell cycle exit curves as well as the advanced dissection of cell cycle fractions revealed that MCF-7nat cells were significantly more responsive to PROTAC than to AMG-232 treatment. The pronounced stop of cell cycle progress, which was seen even in G0/G1-phase of the first cell cycle, indicates that PROTAC disturbs the cell cycle progress even before the cells enter the S-phase or complete a cell cycle. Furthermore, an additional stop in G2/M is introduced by PROTAC. The extra interference at different cell cycle phases makes the PROTAC treatment superior to the use of AMG-232. The difference observed in MCF-7nat cells, however, mostly disappears in MCF-7res cells. In the resistant cells, the treatment with AMG-232 and PROTAC is equivalent efficient. It seems that abemaciclib resistance goes along with a reduced sensitivity to PROTAC in these cells, which makes mdm2 additionally relevant for the CDK4/6-based cell cycle control and for responsiveness to CDK4/6i. The putative lower responsiveness of MCF-7res cells to PROTAC treatment might be due to the slower proliferation capacity in these cells, even though the cells constantly proliferate and remain abemaciclib resistant. However, a PROTAC-induced increase of mdm2 both in CDK4/6i sensitive and resistant MCF-7 cell is associated with a strong anti-tumor effect.

Importantly, p53-mutated T-47D cells respond similar as MCF-7nat cells, which is in agreement with observations made by Adams et al. [42]. The authors impressively demonstrated the usefulness of PROTAC-based mdm2 targeting for the treatment of p53-mutated cells, even though the cells used in their study did not belong to the luminal (i.e., ESR-pos.) cell type. Our study revealed that PROTAC treatment is also very useful and efficient in luminal p53-mutated BC cells as well, whereas the mdm2 inhibitor AMG-232 was practically ineffectual in inhibiting the cell cycle progress in p53 defective cells.

Interestingly, the PROTAC treatment caused a significant generation of apoptotic cell death in regardless of the p53 status. Taken proliferation and apoptosis data together, this indicates that some kind of cell cycle control in p53-mutated T-47D is still active but it is p53-independent.

Unexpectedly, the mdm2 expression in MCF-7 cells increased in the presence of PROTAC, regardless of abemaciclib sensitivity. This might be due to the non-typical PROTAC molecule that consists of two Nutlin molecules [33] and thus does not recruit a non-mdm2 ubiquitin ligase dedicated to ubiquitinate mdm2 as practiced elsewhere [43]. Nevertheless, the PROTAC used here significantly inhibits or even blocks cell proliferation of all cell types subjected to this study, incl. the p53-mutated T-47D cells. However, in contrast to mdm2 in MCF-7 cells and as initially expected, the expression of mdm2 was decreased in T-47D cells, albeit moderately, when exposed to the PROTAC. Obviously, the double-Nutlin-based PROTAC is fairly sufficient in causing mdm2 suicide in p53 mutated cells [33]. To what extent the mutated version of p53 (rather than the wildtype protein) facilitates this process cannot be concluded with certainty at the current state. Importantly, other PROTACs designed to cause mdm2 degradation are not based on two mdm2-ligands but consist of a non-mdm2-ligase instead. For example, “von Hippel-Lindau tumor suppressor” (VHL) or “cereblon” (CRN) ubiquitin ligases are frequently used [31, 44]. CRBN- or VHL-based molecules might initiate mdm2 degradation more efficiently, as seen, for example, by the study presented by Adams et al. [42].

Interestingly, while mdm2 increased in the presence of PROTAC (most likely due to stabilization) in MCF-7 cells the p53 protein was elevated as well. This can be explained by an insufficient binding of p53 to mdm2 in the presence of the double-Nutlin-based PROTAC. This phenomenon disables the ubiquitination of p53 and consequently enables its stabilization. In turn, stabilized p53 causes an increase of mdm2 by a transcriptional feedback-loop [45–48]. More specifically, mdm2 is an essential transcriptional target of p53 so which in turn moderately increases mdm2 under PROTAC treatment. This mechanism, however, cannot be applied to T-47D cells that harbor a defective p53. Instead, p53 remains unaffected in this cell type and because of the loss-of-function a p53 initiated feedback-loop cannot occur. Thus, a p53 mediated stabilization of mdm2 fails in T-47D cells and a PROTAC causes the decrease of mdm2, probably by auto-ubiquitination.

Additionally, a phenomenon previously named the “hook-effect” might be involved in increasing mdm2 in the presence of PROTAC in MCF-7nat and MCF-7res cells. Theoretically, inappropriate PROTAC concentrations (either too low or too high) might stabilize the POI (here mdm2). Instead, the PROTAC could act as a “molecular glue” and might recruit neo-substrates for their ubiquitination and subsequent degradation, while the degradation of mdm2 would become inefficient or even could not take place anymore [32]. However, based on the initially performed concentration series, we assume that the “hook effect” most likely plays a minor role. In addition, it can be assumed that using the lowest PROTAC concentration that elicits a maximum efficiency is useful to minimize (or almost exclude) off-target effects.

Notably, in this study we evidence a p53-independent mechanism of PROTAC. This is supported by analyses done by Klein et al., who showed that mdm2 plays a p53-independent role in maintaining cell cycle progression, e.g., by promoting the activity of E2F1 factors as well as p73 [49]. Our study is in agreement with that finding because p53-mutated T-47D and p53 wildtype MCF-7nat cells show a similar and remarkable treatment response to PROTAC. This consideration is compatible with the findings again reported by Adams et al. [42], who demonstrated that triple negative breast cancer cells - when exposed to PROTAC - show elevated p73 that takes over the tumor suppressor activity of an inactive p53. Even though not yet been elucidated in detail, p73 is known to have the capacity to compensate for the lost p53 function. This can be explained by a substantial overlap of p53 und p73 transcription targets [50], e.g., p21 (otherwise referred to as “Cyclin Dependent Kinase Inhibitor 1A” CDKN1A), mdm2, and BAX [51]. Strikingly, and in contrast to the observation made by Adams et al., in our study the PROTAC treatment caused a significant decrease and a nearly complete elimination of p73 in T-47D and abemaciclib sensitive and resistant MCF-7 cells. In this context it is important to realize that the study by Adams et al. is based on triple-negative but non luminal (i.e., ESR-pos.) BC cells. It is quite certain that the cognate molecules p53 and p73 (and potentially p63) underlie a disparate regulation in the presence and absence of the ESR, respectively [52, 53]. Although p53 and p73 share a significant number of transcription targets, it is also known that p73 may not necessarily be p53’s natural substitute in enforcing tumor suppression [51]. Indeed, the effects of p73 seem to differ in triple-negative and ESR-pos. (BC) cells. Consequently, the PROTAC treatment efficiency is not necessarily p73-dependent, not even in p53-mutated cells. Nonetheless, the achieved treatment effect by PROTAC seems to be based on a p53 bypass, most likely both in p53 wildtype and mutated cells.

Interestingly, silencing (but not degradation) of mdm2 has been associated with reduced endogenous p73 expression, but again, not BC but other malignant cells were used [54]. This comparison repeatedly suggests that different strategies of mdm2 inactivation (inhibition, silencing, degradation) and the use of different target cells uncovers context-specific effects on the expression and presence of p73. Extending research should explicitly address the ESR activity and its transcriptional output under PROTAC treatment. Further exploration should include Cyclin-D1, which is a highly relevant transcriptional target of the ESR in luminal BC. As a main regulator of CDK4/6 it plays a crucial role in ESR-pos. BC, in particular when treated with CKDK4/6i.

A common finding derived from MCF-7nat and MCF7res cells is the substantial time-dependent increase of p21 in the presence of PROTAC, which is not observable in T-47D cells. p21 seems to play a pivotal role in exhibiting a PROTAC treatment response in p53 wildtype cells but rather not in p53-mutated cells. p21 is known not only to be involved in the DNA damage response [55] but also in the cell cycle control. It is being known for a long time that p21, as member of the “Interacting Protein/Kinase inhibitory proteins” (CIP), inhibits a variety of CDKs, amongst them CDK2, 3, 4, and 6 [56–58]. Interestingly, both tumor-suppressive but also potential oncogenic functions have been attributed to p21 [15, 55, 59], whereas p21 obviously exhibits its suppressive activity under the influence of PROTAC. Thus, the PROTAC-induced increase of p21 explains the considerable deceleration of the cell cycle progress, in particular in MCF-7 p53 wildtype cells.

p21 represents a central negative-regulator of pro-proliferative proteins transcribed by the E2F family, which in turn is tightly controlled by the Rb protein [60]. By forming a complex with Cyclin-D/CDK4/6 p21 prevents the phosphorylation of Rb, which disables the transcriptional activity of E2F. Alternatively, p21 can indirectly prevent the Rb phosphorylation and the subsequent E2F1 release by interacting with the Cyclin-E/CDK2 complex [15]. Notably, p21 represents key transcriptional target of p53 [60]. Accordingly, it can be suggested that in the presence of PROTAC the already present p21 gets stabilized rather than the expression increased.

The pronounced presence and activity of p21 under PROTAC treatment correlates with a considerable decrease and nearly complete eradication of total and phosphorylated Rb levels in all three cell types. We analyzed four different Rb phosphorylation sites (i.e., Ser608, Ser780, Ser795, and Ser807/811). All of them are known to contribute to the E2F1 release and to enable its transcriptional activity, even though not all by inducing an Rb conformational change but also by alternative mechanisms [61, 62]. Both the repression of Rb expression and its reduced phosphorylation in PROTAC-treated MCF-7 and T-47D cells can be considered to act as the ultimate event that blocks (or at least considerably decelerates) the cell cycle progress. Notably, this event occurs equally in all three cell lines, regardless of initial PROTAC-triggered molecular effects. This suggests that different initially PROTAC-induced upstream events can result in identical downstream modifications, which finally curb cell proliferation. Additional molecular analyses are warranted to further elucidate the molecular mechanism, which are triggered by mdm2 PROTAC degradation.

For comparison purposes, we also exposed the cells to the mdm2 inhibitor AMG-232 and analyzed molecular effects over time. The most striking difference of alterations compared to those seen in PROTAC-treated cells is that mdm2 increased in all three cell types in the presence of AMG-232, even though the effect was rather transient in particular in the less responsive MCF-7res and not-responsive T-47D cells. However, the p73 was basically unaffected in MCF-7nat cells and even a slight stabilization could be observed in T-47D cells. Also important to note is, that the increase of p21 in T-47D cells is unsustainable and, in contrast to MCF-7 cells, a decrease of E2F1 does virtually not occur. These findings are compatible with the resistance of T-47D cells to the mdm2 inhibitor AMG-232 (see proliferation data) and are only seen in the p53-mutated cell type.

Thus, different molecular effects become obvious in PROTAC- and AMG-232-treated cells, respectively, even though the treatment with both substances inhibit cell proliferation of the two MCF-7 cell types. Nevertheless, the administration of PROTAC revealed superior efficiency. Even more important, T-47D cells are insensitive to AMG-232, which underlines the greater effects triggered by PROTAC and its usability for treatment of cells with p53 mutation. In contrast, we (and others) observed severe cytotoxic effects of AMG-232 treatments both in-vitro and in-vivo, which hampers the translation to a clinical application [12, 63]. In comparison, potential PROTAC-induced cytotoxic and other side effects on normal (i.e., non-malignant) cells and tissues can be best prospectively assessed by preclinical in-vivo studies using an appropriate mouse model (see also below).

The PROTAC-triggered molecular effects observed in both MCF-7 cell types and in T-47D cells are translated into a functional model illustrated in supplement Fig. 3. Common and different mechanisms become apparent. Even though molecular mechanisms differ to some extent, the PROTAC treatment is highly efficient and is superior to mdm2 inhibition in MCF-7nat and T-47D cells.

Beyond the analysis of the molecular intracellular signaling, we analyzed the PROTAC-induced effect on the immunogenicity of target cells. It has been reported that an inactivation of p53 by the mdm2 ligand Nutlin-3 causes the significantly elevated expression of the immune blockade receptors PD-L1 and CD276 [64]. Moreover, it was found that the two inhibitory paralog receptors are under different genetic control. More specifically, the induction of CD276 was p53-dependent, whilst PD-L1 was elevated upon Nutlin treatment even in p53-null cells. Cellular (side) effects of an mdm2 targeting on immune-checkpoint expression is potentially relevant as they have the capacity to curb or even to block an endogenous immunological tumor defense. Similar to PD-L1, CD276 prevents the surveillance of tumor cells by cytotoxic T and NK-cells [65] and its expression on BC cells has been reported to associate with poor prognosis, enlarged tumor size, lymph node migration and cancer recurrence [66, 67]. In contrast to the observation made by Li et al., we observed here a significantly reduced CD276 and MHC-I expression in PROTAC-treated MCF-7nat, MCF-7res, and T-47D cells, while the cell exposition to AMG-232 had no effect on these checkpoints. Furthermore, we found that PD-L1, PD-L2, and MHC-II were not affected in the presence of PROTAC. A reduced MHC-I expression would on the one hand impede the recognition by cytotoxic T cells. On the other hand, lowered MHC-I would result in a *“missing-self”* phenotype of tumor cells, which could release the brake on NK cells that would elicit a tumor defense by the innate immune cells [68, 69]. The mechanism that underlies MHC-I downregulation in the presence of PROTAC is unknown, even though Massafra et al., who applied bromo- and extraterminal peptide-directed PROTACs observed similar effects [70]. In contrast, decreased CD276 could reduce immune-suppressive effects by tumor cells. However, the presence of immune activating molecules on luminal BC cells is inherently rather low or does basically not exist [71], which means that a PROTAC-induced (further) decrease of CD276 and MHC-I would most likely not additionally alter the immunological invisibility of luminal BC cells. Nevertheless, the effects of PROTAC-treated tumor cells on immune cells remain to be explored by using appropriate examination models. The use of humanized tumor mice, for example, could noticeably promote those analyses [72, 73].

## Conclusion

Overall, the here presented in-vitro analyses revealed highly effective PROTAC-based mdm2 targeting of ESR-pos. BC cells both with and without functional p53. Moreover, abemaciclib-resistant MCF-7 cells retain their sensitivity to the mdm2 targeting, in particular when PROTAC is used. The double-Nutlin-based PROTAC does not necessarily result in a reduced mdm2 expression level. Further downstream, other molecular alterations triggered by the PROTAC administration differ in p53 wildtype and mutated cells but have a substantial downregulation and inactivation of p73 and Rb in common. Cell type-specific PROTAC-initiated molecular mechanisms with severe impact on cell proliferation and survival in ESR-pos. cells need to be deciphered in more detail. It appears worthwhile to further pursue with the development and testing of highly specific PRTOAC molecules against mdm2 (and other target molecules) to overcome limitations of small-molecule inhibitors (e.g., anti-CDK4/6). Moreover, PROTAC-based molecule degradation allows to re-consider previously undruggable targets and pathways, for example those affected by a functionally defective tumor suppressor p53. Further preclinical analyses using appropriate in-vivo models are essential to push PROTAC developments and to enable their transfer into clinical practice.

## Electronic supplementary material

Below is the link to the electronic supplementary material.

Supplementary Material 1

Supplementary Material 2

Supplementary Material 3

Supplementary Material 4

## Data Availability

The datasets used and/or analysed during the current study are available from the corresponding author on reasonable request.

## Declarations

### Use of artificial intelligence tools

AI tools were not used.
