# Supplementary material for: Mdm2 targeting via PROteolysis TArgeting Chimeras (PROTAC) is efficient in p53 wildtype, p53-mutated, and abemaciclib-resistant estrogen receptor-positive cell lines and superior to mdm2 inhibition
Source: BMC Cancer. 2025 Jun 1;25:978. doi: 10.1186/s12885-025-14361-z (PMC12128487; doi:10.1186/s12885-025-14361-z)
Supplement: Supplementary file 1 — Supplementary Material 1 [file 12885_2025_14361_MOESM1_ESM.pdf]

Selection of uncropped Western Blots (Fig. 3 and 4)

Please note:

- Only one selected data set is shown in this compilation, respectively, however, all experiments were performed three times.
- Western Blot bands in red indicate **overexposure** for that particular band. This indicates differences in protein concentration. Nevertheless, individual bands (e.g., actin as control and protein of interest) were developed separately under optimized exposure conditions.

MCF-7nat

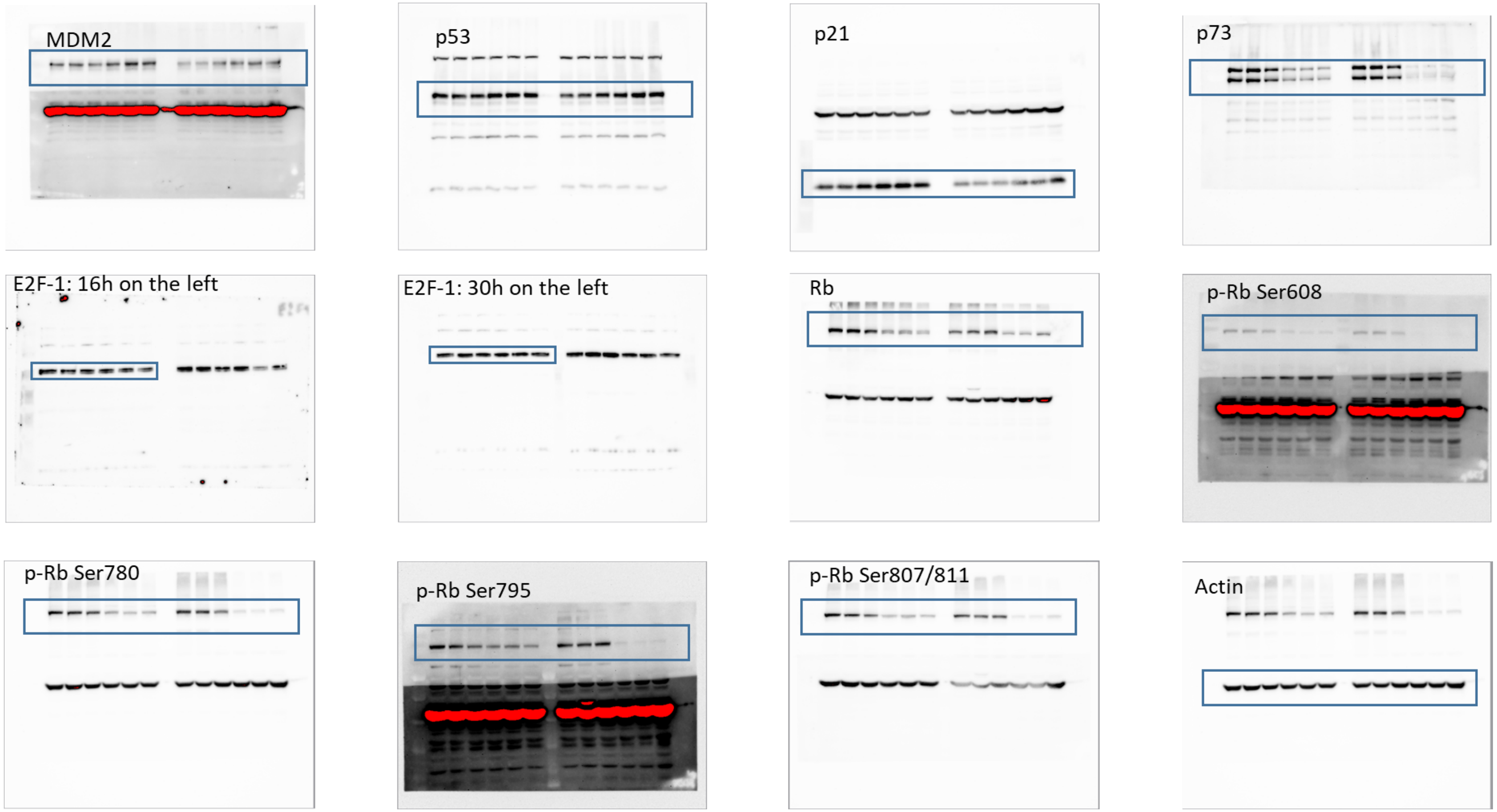

This selected example refers to Figure 3

Intracellular signaling in MCF-7nat, MCF-7res, and T-47D cells as a function of PROTAC treatment with increasing concentrations. Cells were treated for 16 and 30 h with 2.5, 5.0, 10, and 20 µM PROTAC before cells lysis and protein isolation. Completely untreated and DMSO-treated (PROTAC solvent) cells served as negative controls. Representative Western Blots of mdm2, p53, p73, p21, E2F1, Rb, pRbSer608, pRbSer780, pRbSer795, and pRbSer807/811 are shown.

MCF-7res

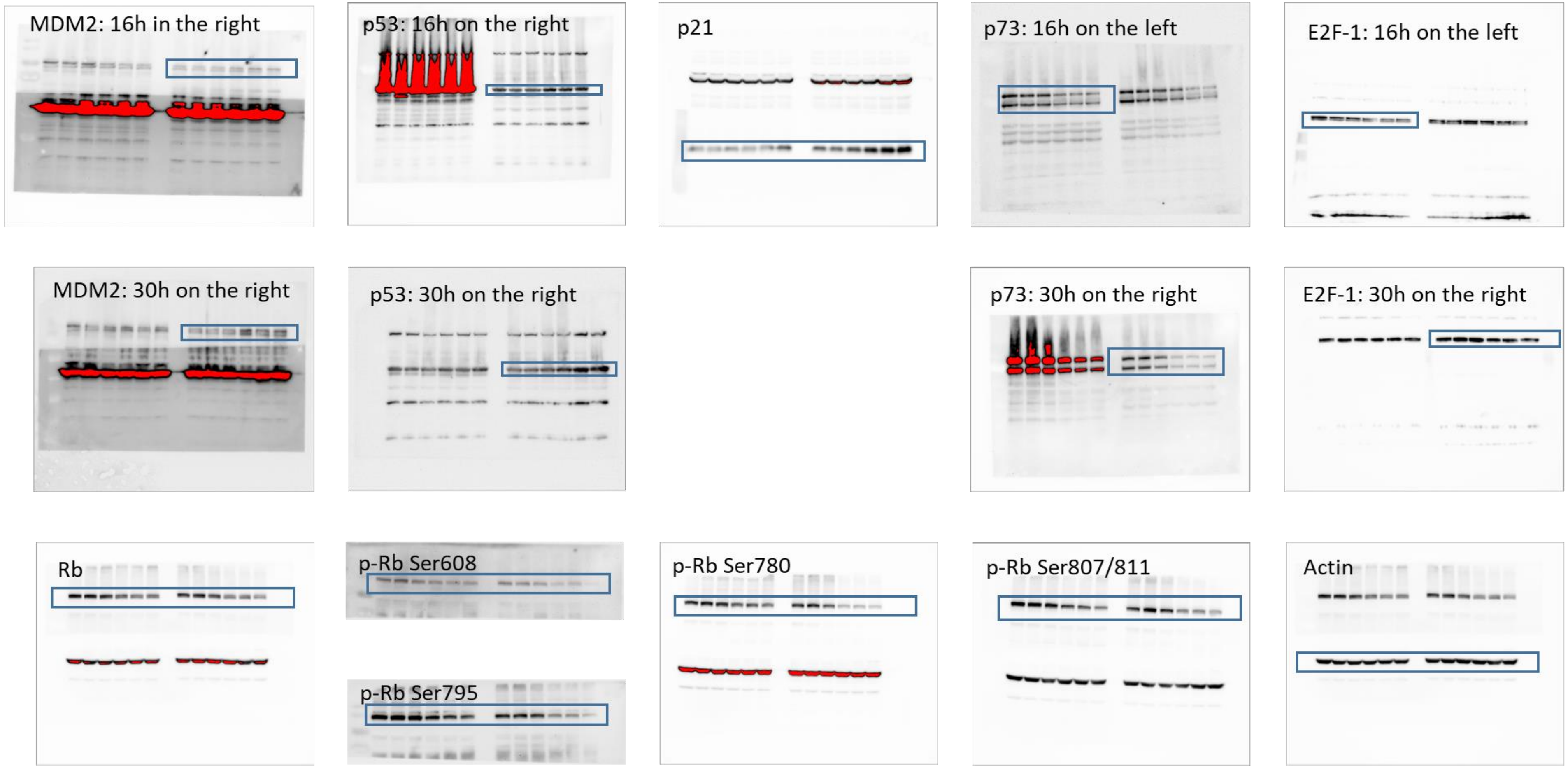

This selected example refers to Figure 3

Intracellular signaling in MCF-7nat, MCF-7res, and T-47D cells as a function of PROTAC treatment with increasing concentrations. Cells were treated for 16 and 30 h with 2.5, 5.0, 10, and 20 µM PROTAC before cells lysis and protein isolation. Completely untreated and DMSO-treated (PROTAC solvent) cells served as negative controls. Representative Western Blots of mdm2, p53, p73, p21, E2F1, Rb, pRbSer608, pRbSer780, pRbSer795, and pRbSer807/811 are shown.

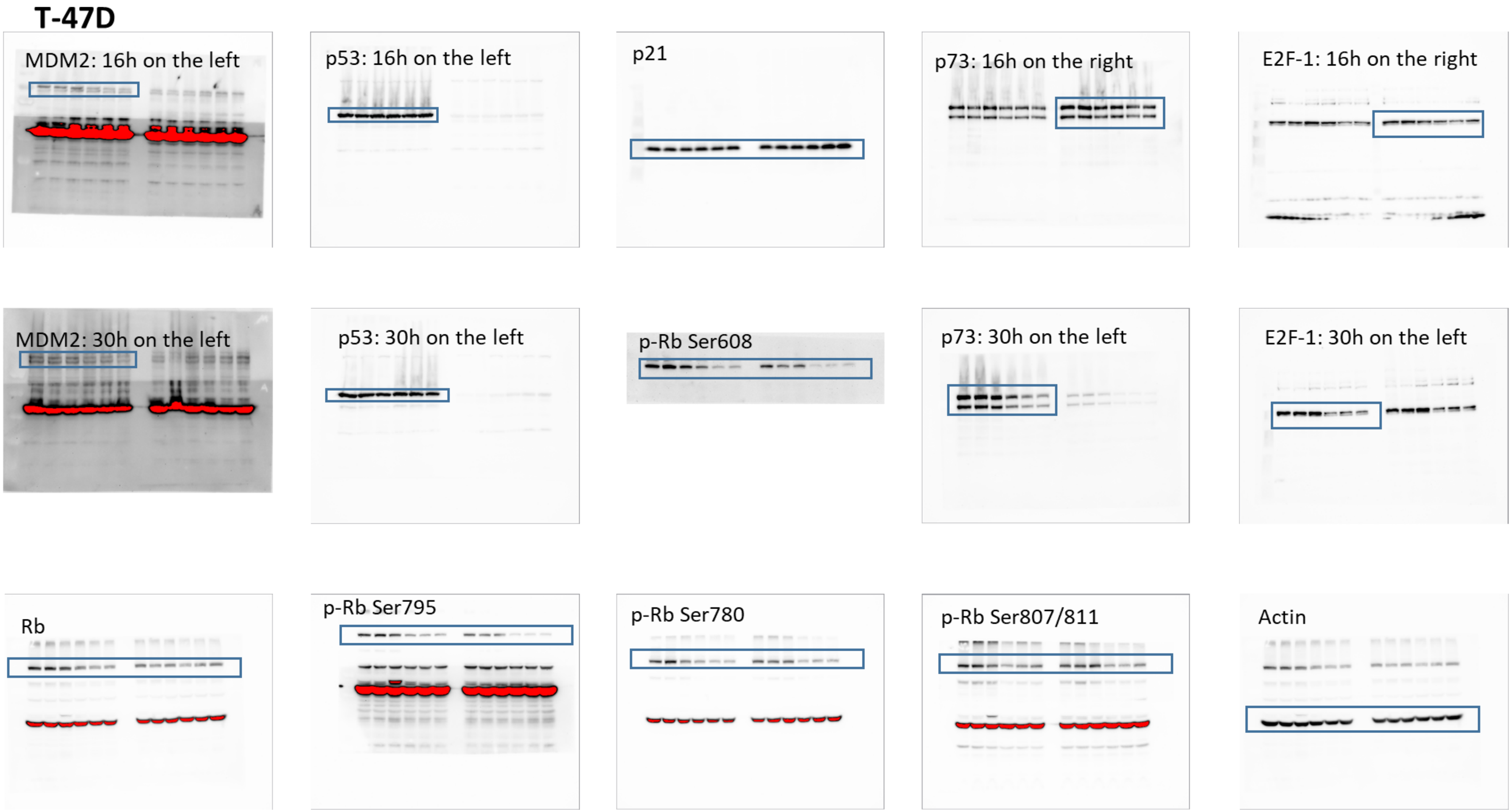

This selected example refers to Figure 3

Intracellular signaling in MCF-7nat, MCF-7res, and T-47D cells as a function of PROTAC treatment with increasing concentrations. Cells were treated for 16 and 30 h with 2.5, 5.0, 10, and 20 µM PROTAC before cells lysis and protein isolation. Completely untreated and DMSO-treated (PROTAC solvent) cells served as negative controls. Representative Western Blots of mdm2, p53, p73, p21, E2F1, Rb, pRbSer608, pRbSer780, pRbSer795, and pRbSer807/811 are shown.

MCF-7nat

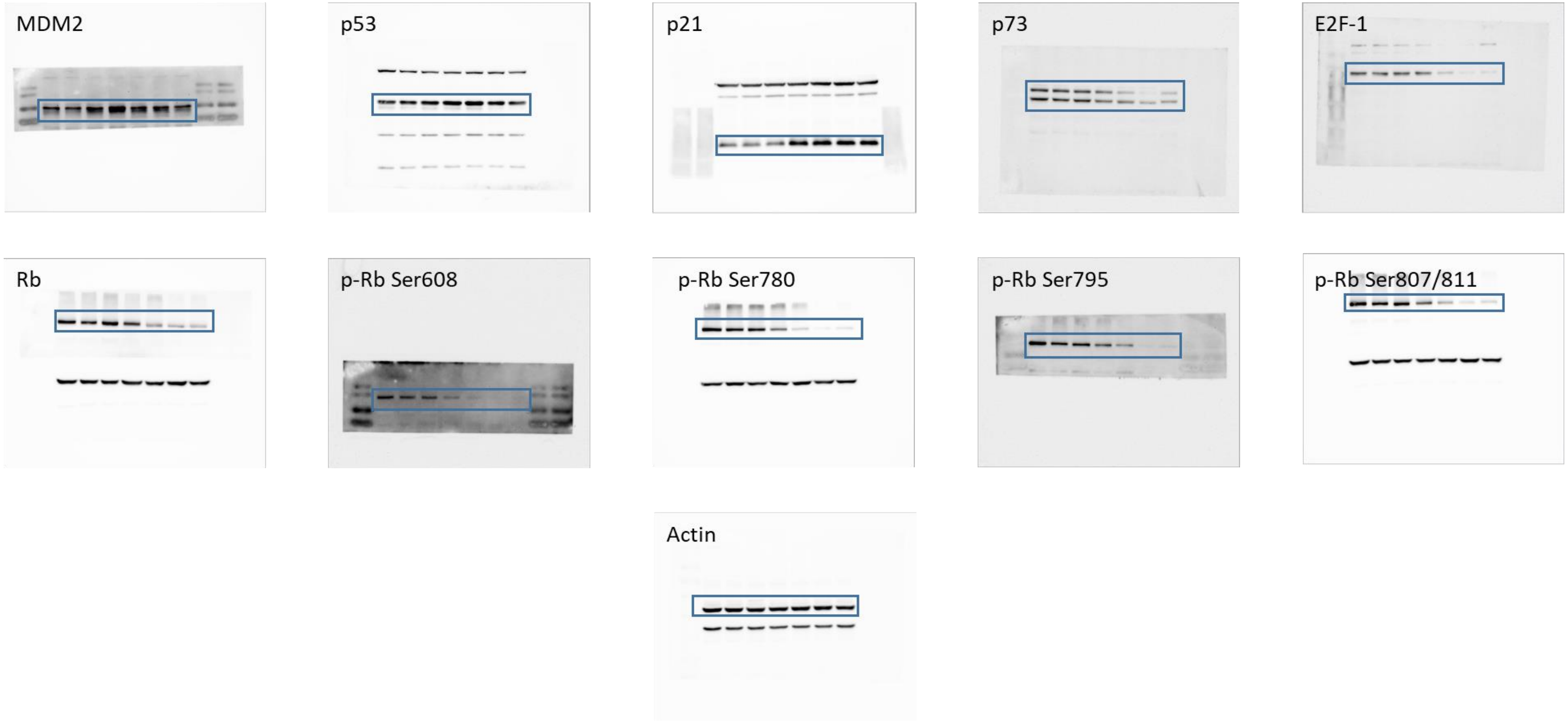

This selected example refers to Figure 4

Intracellular signaling in MCF-7nat, MCF-7res, and T-47D cells as a function of AMG-232 treatment over a period of 48 h. Samples taken after 30 min, 2, 8, 16, 30, and 48 h intervals are shown. mdm2, p53, p73, p21, E2F1, Rb, pRbSer608, Ser780, Ser795, and Ser807/811 were analyzed.

MCF-7res

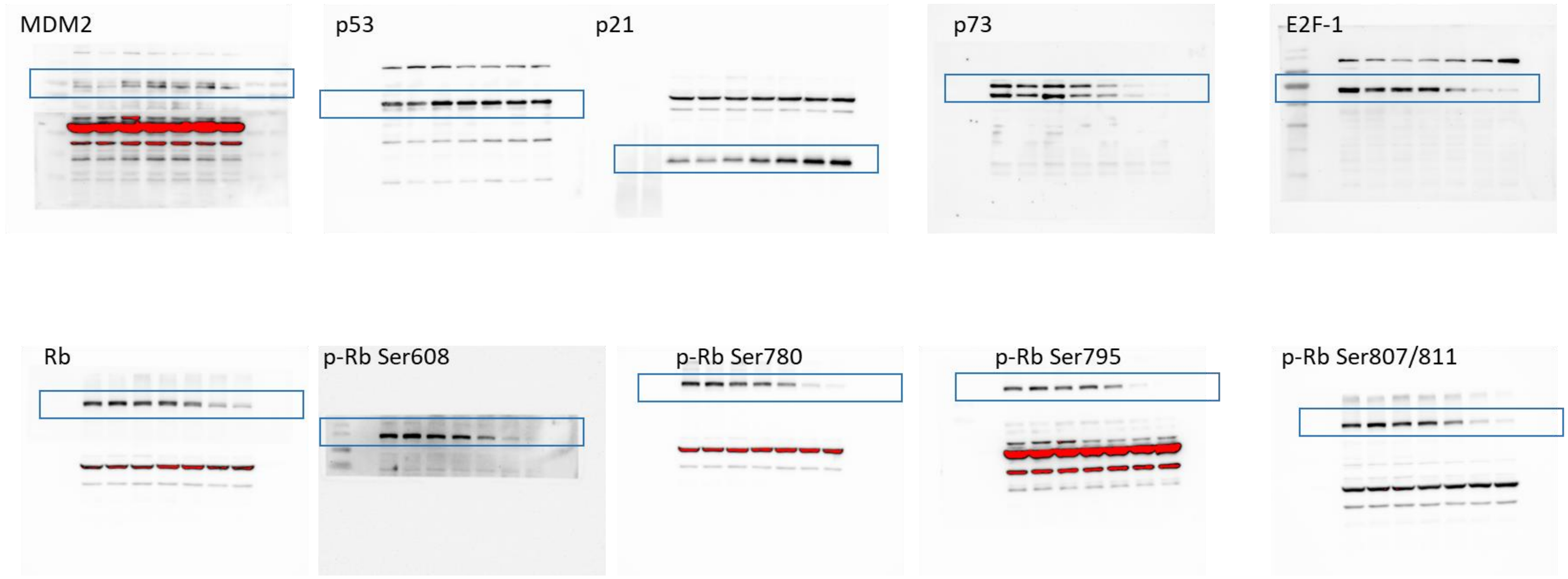

This selected example refers to Figure 4

Intracellular signaling in MCF-7nat, MCF-7res, and T-47D cells as a function of AMG-232 treatment over a period of 48 h. Samples taken after 30 min, 2, 8, 16, 30, and 48 h intervals are shown. mdm2, p53, p73, p21, E2F1, Rb, pRbSer608, Ser780, Ser795, and Ser807/811 were analyzed.

T-47D

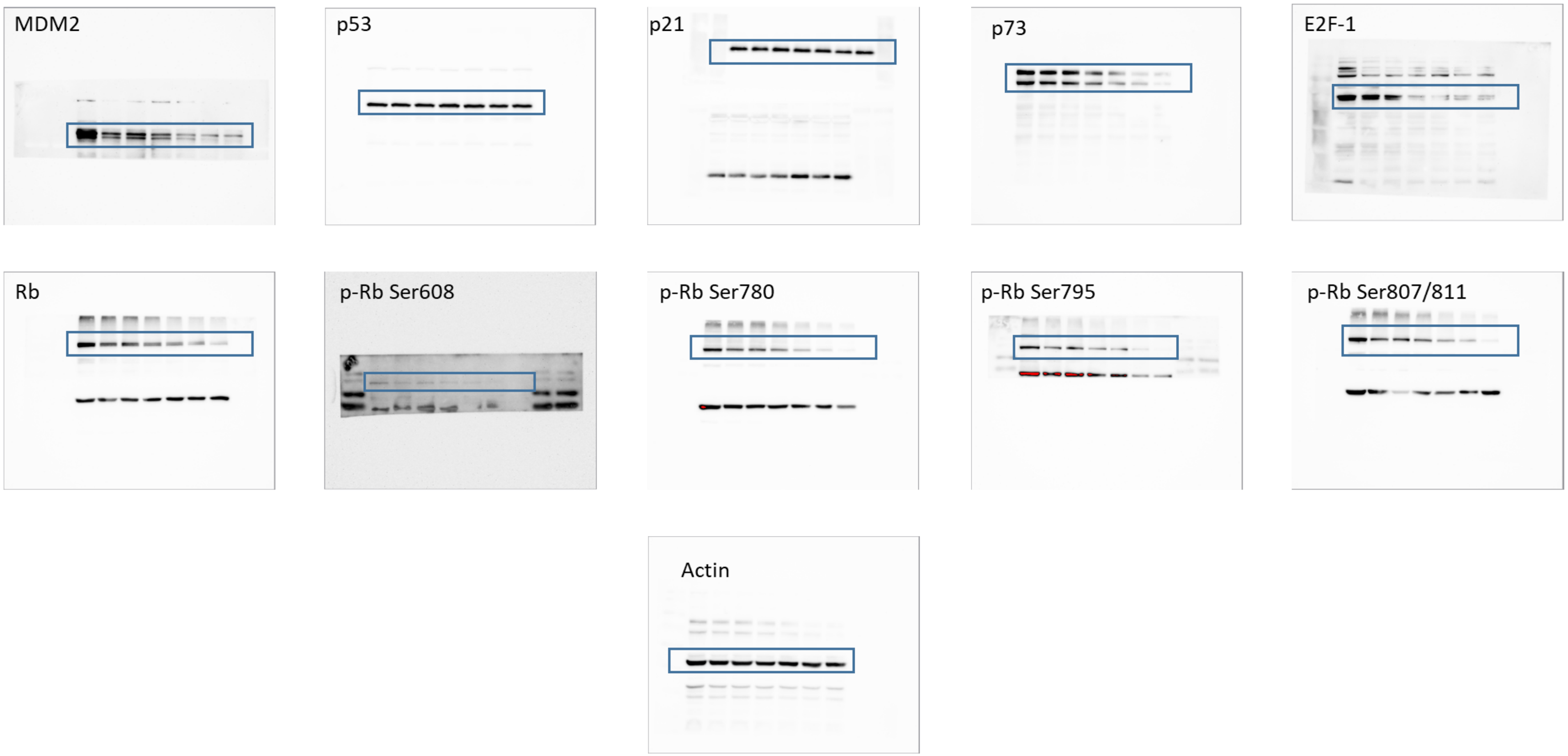

This selected example refers to Figure 4

Intracellular signaling in MCF-7nat, MCF-7res, and T-47D cells as a function of AMG-232 treatment over a period of 48 h. Samples taken after 30 min, 2, 8, 16, 30, and 48 h intervals are shown. mdm2, p53, p73, p21, E2F1, Rb, pRbSer608, Ser780, Ser795, and Ser807/811 were analyzed.
