# Supplementary figures and images for: Mdm2 targeting via PROteolysis TArgeting Chimeras (PROTAC) is efficient in p53 wildtype, p53-mutated, and abemaciclib-resistant estrogen receptor-positive cell lines and superior to mdm2 inhibition
Source: BMC Cancer. 2025 Jun 1;25:978. doi: 10.1186/s12885-025-14361-z (PMC12128487; doi:10.1186/s12885-025-14361-z)

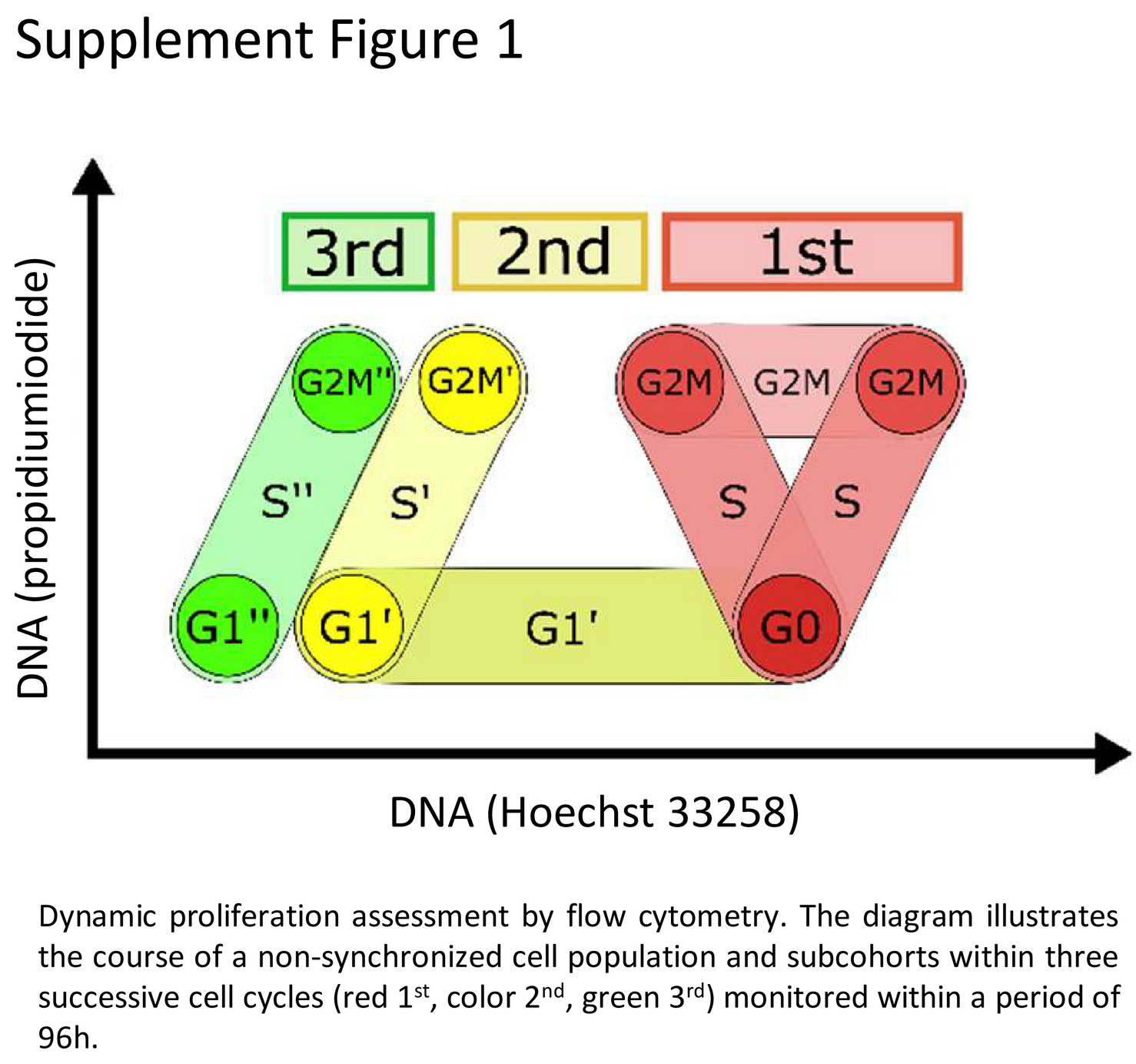

Supplement: Supplementary file 2 — Supplementary Material 2 [file 12885_2025_14361_MOESM2_ESM.tif]

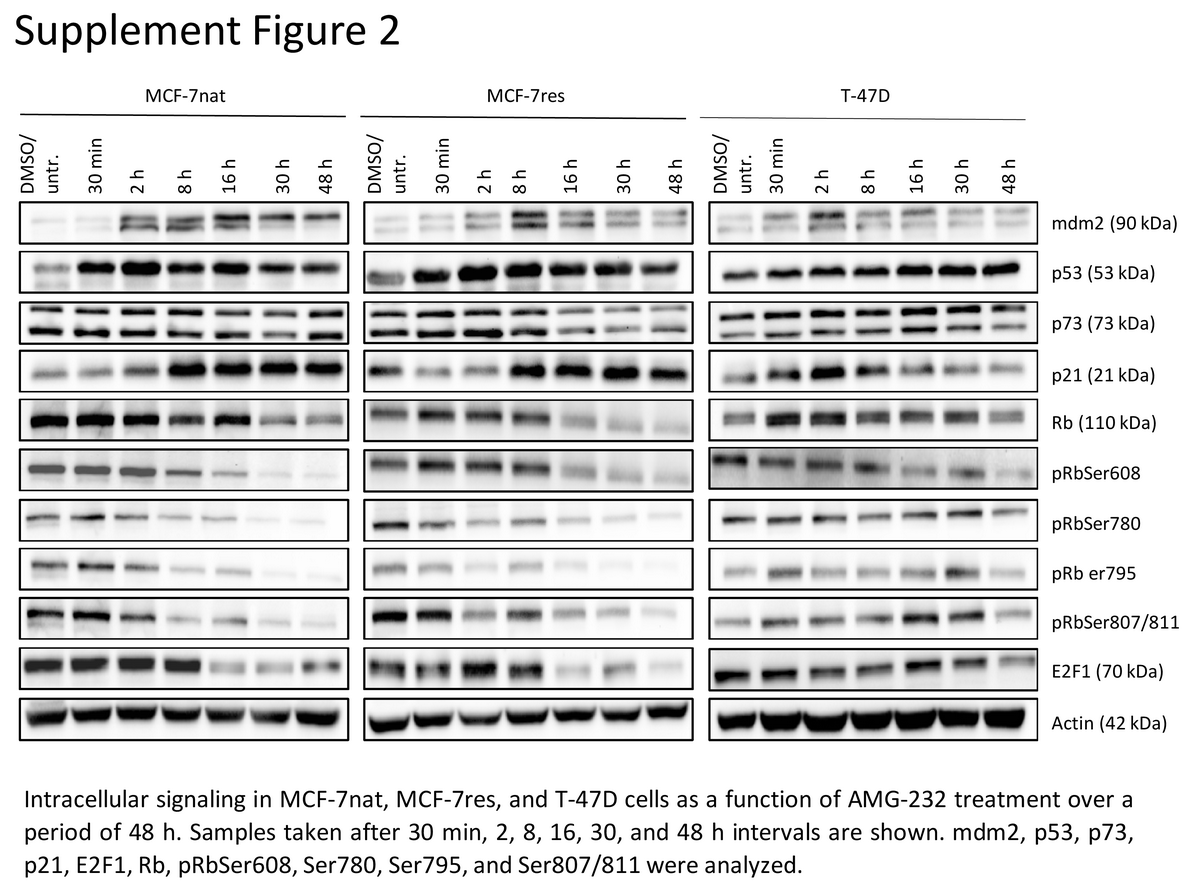

Supplement: Supplementary file 3 — Supplementary Material 3 [file 12885_2025_14361_MOESM3_ESM.tif]

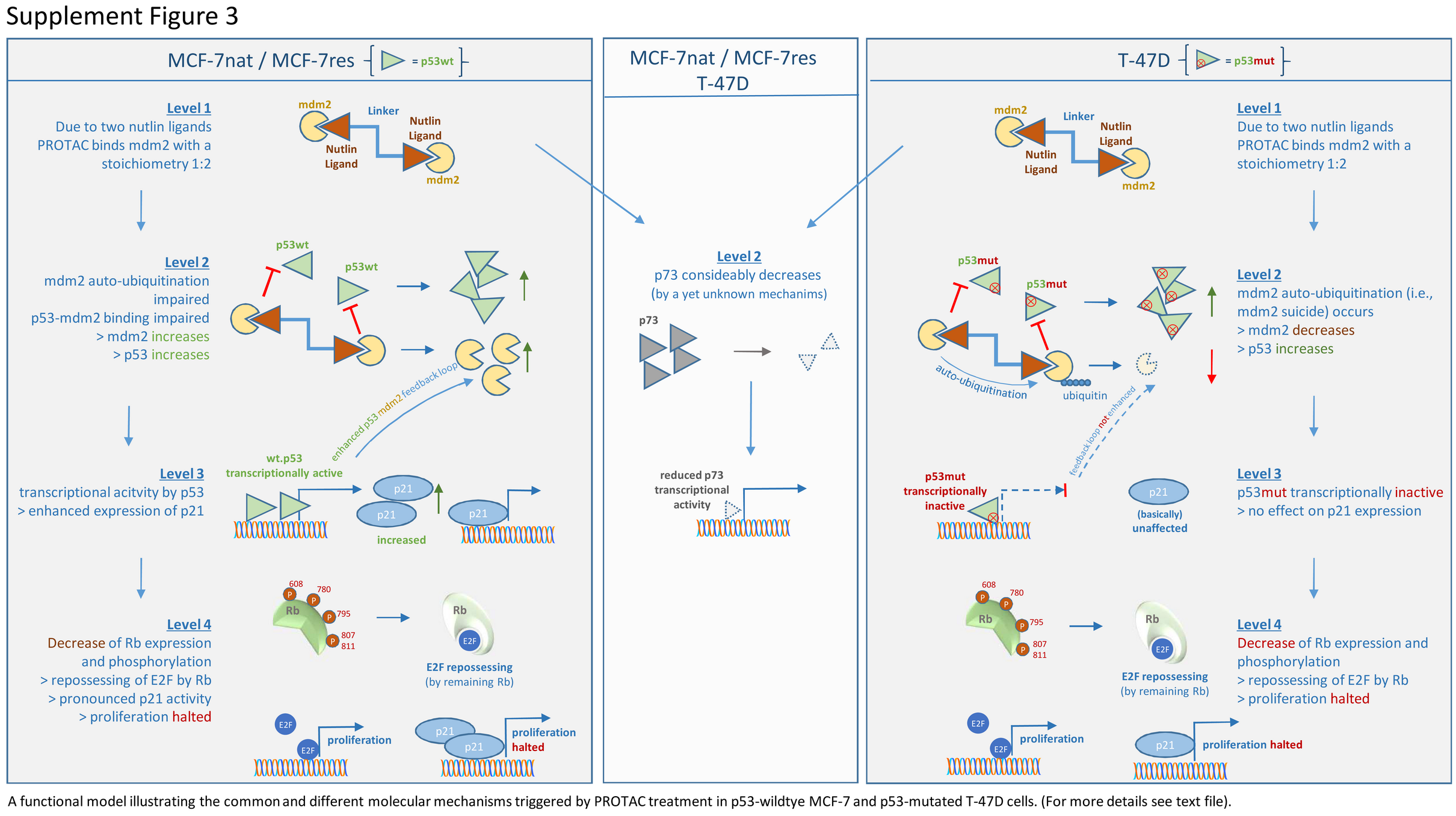

Supplement: Supplementary file 4 — Supplementary Material 4 [file 12885_2025_14361_MOESM4_ESM.tif]
